# Inhibition of Voltage-Gated Sodium Channels by Animal Toxins

**DOI:** 10.1063/4.0000981

**Published:** 2025-10-27

**Authors:** Shane Gonen

**Affiliations:** UC Irvine, Irvine, CA, USA

## Abstract

Voltage-gated sodium channels (NaVs) play important roles in propagating action potentials and their structure and function can be modulated by a variety of factors, including animal toxins. Despite recent technological advances, channel and toxin structural studies still present many challenges. We used cryogenic electron microscopy (CryoEM) to study the tetrodotoxin-resistant human NaV 1.8 at high resolution and as a complex with a peptide derived from the Peruvian green velvet tarantula, Protoxin-I. We describe our methodology to bypass biochemical challenges and discuss the inhibition of the channel by the binding of Protoxin-I. We also gained information on a class of animal toxins derived from cone snails on a larger scale using both comparative and deep-learning modeling methods. These results give us a clearer picture of the interactions of animal toxins with NaVs.

